# Genetic Deletion of Vasohibin-2 Exacerbates Ischemia-Reperfusion-Induced Acute Kidney Injury

**DOI:** 10.3390/ijms21124545

**Published:** 2020-06-26

**Authors:** Hiromasa Miyake, Katsuyuki Tanabe, Satoshi Tanimura, Yuri Nakashima, Tomoyo Morioka, Kana Masuda, Hitoshi Sugiyama, Yasufumi Sato, Jun Wada

**Affiliations:** 1 Department of Nephrology, Rheumatology, Endocrinology and Metabolism, Okayama University Graduate School of Medicine, Dentistry and Pharmaceutical Sciences, Okayama 7008558, Japan; pijk3kjj@s.okayama-u.ac.jp (H.M.); qinokospraut@gmail.com (S.T.); straw126g3reen@gmail.com (Y.N.); mundo.feliz330@gmail.com (T.M.); kana71love@hotmail.com (K.M.); junwada@okayama-u.ac.jp (J.W.); 2 Department of Human Resource Development of Dialysis Therapy for Kidney Disease, Okayama University Graduate School of Medicine, Dentistry and Pharmaceutical Sciences, Okayama 7008558, Japan; hitoshis@okayama-u.ac.jp; 3 New Industry Creation Hatchery Center, Tohoku University, Sendai 9808575, Japan; yasufumi.sato.b3@tohoku.ac.jp

**Keywords:** acute kidney injury, ischemia-reperfusion, oxidative stress, vasohibin-2, peritubular capillaries

## Abstract

Acute kidney injury (AKI) has been increasingly recognized as a risk factor for transition to chronic kidney disease. Recent evidence suggests that endothelial damage in peritubular capillaries can accelerate the progression of renal injury. Vasohibin-2 (VASH2) is a novel proangiogenic factor that promotes tumor angiogenesis. However, the pathophysiological roles of VASH2 in kidney diseases remain unknown. In the present study, we examined the effects of VASH2 deficiency on the progression of ischemia–reperfusion (I/R) injury-induced AKI. I/R injury was induced by bilaterally clamping renal pedicles for 25 min in male wild-type (WT) and *Vash2* homozygous knockout mice. Twenty-four hours later, I/R injury-induced renal dysfunction and tubular damage were more severe in VASH2-deficient mice than in WT mice, with more prominent neutrophil infiltration and peritubular capillary loss. After induction of I/R injury, VASH2 expression was markedly increased in injured renal tubules. These results suggest that VASH2 expression in renal tubular epithelial cells might be essential for alleviating I/R injury-induced AKI, probably through protecting peritubular capillaries and preventing inflammatory infiltration.

## 1. Introduction

Acute kidney injury (AKI) is an abrupt loss of kidney function, which is characterized by increased serum creatinine concentration and decreased urine volume within seven days [1]. AKI has traditionally been recognized as a “transient” disorder; that is, patients with AKI should show complete recovery of their renal function. However, accumulating evidence suggests that AKI can subsequently result in the onset of chronic kidney disease (CKD) in some patients [2] and accelerate the progression of underlying CKD in other patients [3]. Therefore, AKI demonstrates poorer outcomes than previously reported. Since the pathophysiology of AKI has not yet been fully elucidated, no effective therapeutic strategies against AKI have been established.

Ischemia–reperfusion (I/R) injury is one of the most common etiology of hospital-acquired AKI. Although I/R injury is typically accompanied with partial nephrectomy or kidney transplantation, it has also been recognized to be associated with renal hemodynamic instability, such as sepsis [4]. Local ischemia results in an imbalance between oxygen supply and demand in the renal tubular epithelium, and reperfusion induces oxidative stress, leading to apoptosis and necrosis of tubular epithelial cells. The renal outer medulla and corticomedullary boundary are most susceptible to I/R injury because of low blood supply relative to high oxygen demand in the area. Recent evidence has revealed the critical roles of endothelial damage and dysfunction in renal I/R injury [5]. Endothelial dysfunction not only reduces local blood flow to the tubular epithelium but also enhances infiltration of inflammatory cells, mainly neutrophils, into the renal interstitium. Previous studies reported that endothelial nitric oxide synthase (NOS) knockout or NOS inhibitor administration exacerbated the degree of renal tubular damage caused by renal I/R injury [6,7].

Peritubular capillaries (PTCs) provide oxygen and nutrients to the renal tubules, and ischemic insult decreases the density of PTC network [8]. The representative proangiogenic factor, vascular endothelial growth factor (VEGF), is known to be important for maintaining the structure of the PTC network. It is secreted by glomerular and, to a lesser extent, tubular epithelial cells and acts on its receptors expressed on glomerular and PTC endothelial cells, respectively [9]. The critical roles of VEGF in the PTC network have been demonstrated by a recent study, which showed a marked reduction in the PTC area in renal tubule-specific VEGF knockout mice [10]. Furthermore, previous studies reported that renal VEGF expression was repressed by I/R injury [11], and administration of VEGF-121 preserved PTC density in post-I/R kidneys [12], suggesting the potential protective effects of VEGF on the PTC network in renal I/R injury.

Vasohibin-2 (VASH2) is a novel proangiogenic protein. It was originally identified as a homologue to vasohibin-1 (VASH1) [13], which is a negative-feedback regulator of angiogenesis. VASH2 expression is known to be upregulated in cancer cells and promotes cancer angiogenesis as well as growth and metastasis [14]. Moreover, recent reports demonstrated that VASH2 induced epithelial-to-mesenchymal transition (EMT) in cancer cells, leading to higher malignancy potential [15]. Although the significance of VASH2 expression in cancer has become increasingly apparent, the physiological roles of VASH2 in any organs, including the kidney, remain to be elucidated. Our recent study demonstrated that VASH2 expression in the kidney was increased in diabetic mice [16]. Furthermore, immunohistochemistry for VASH2 in kidney specimens from patients with CKD revealed increased renal tubular VASH2 staining compared with control specimens [17]. These findings suggest that VASH2 may be upregulated in the kidney in response to a variety of insults and involved in renal pathological processes.

In the present study, we examined the pathophysiological significance of VASH2 expression in I/R-induced AKI using VASH2 knockout mice. Genetic deletion of VASH2 resulted in more severe renal dysfunction and tubular injury as well as PTC loss and neutrophil infiltration. Furthermore, renal VASH2 expression in wild-type (WT) mice was upregulated by I/R injury. Our results suggest that increased renal expression of VASH2 in I/R injury may protect the renal tubular epithelium through protecting PTCs and preventing inflammatory infiltration.

## 2. Results

### 2.1. VASH2 Deficiency Accelerated I/R-Induced Renal Tubular Injury

**Figure 1:**
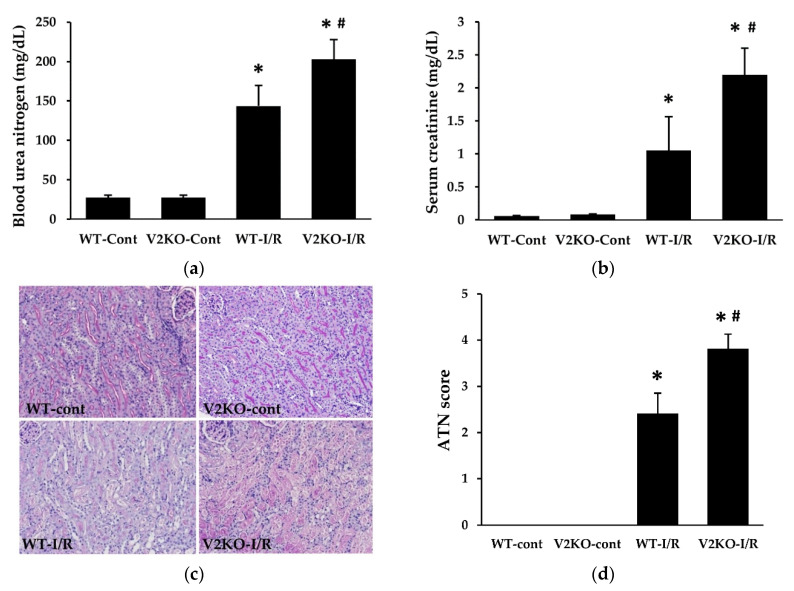
Vasohibin-2 (VASH2) deficiency accelerated ischemia–reperfusion (I/R)-induced renal dysfunction and renal tubular injury. The increase in serum levels of blood urea nitrogen (BUN) (**a**) and creatinine (**b**) induced by I/R injury was higher in V2KO mice than WT mice; (**c**) light microscopy images of the renal corticomedullary areas (periodic acid-Schiff (PAS) staining, original magnification, ×200). (**d**) Acute tubular necrosis (ATN) score was calculated based on the percentage of damaged tubules. *n* = 6 for each group. * *p* < 0.01 vs. WT-cont, ^#^ *p* < 0.05 vs. WT-I/R. Each column shows the mean ± standard deviation (SD).

In accordance with a previous report [16], compared with WT mice, *Vash2* homozygous knockout (*Vash2*^LacZ/LacZ^; V2KO) mice did not show any changes in phenotypes, including renal function and morphology (Figure 1). I/R injury was induced in WT and V2KO mice (WT-I/R and V2KO-I/R, respectively), and control mice received sham operation (WT-cont and V2KO-cont, respectively). Although there was no difference in body weight between WT-I/R and V2KO-I/R mice (19.6 ± 1.0 vs. 20.5 ± 2.1 g), the kidney-weight-to-body-weight ratio in V2KO-I/R mice (7.4 ± 0.6 mg/g) tended to be higher than that in WT-I/R mice (6.8 ± 0.8 mg/g). I/R injury significantly increased blood urea nitrogen (BUN) and serum creatinine levels in both WT and V2KO mice. However, the increase was more prominent in V2KO-I/R than WT-I/R mice (Figure 1a,b). I/R-induced renal tubular injury, including epithelial cell detachment, brush border loss, and intraluminal cast formation was assessed using periodic acid-Schiff (PAS)-stained specimens (Figure 1c) and quantified as acute tubular necrosis (ATN) score. Increased ATN score caused by I/R injury was significantly higher in V2KO-I/R mice than in WT-I/R mice (Figure 1d). These results suggested that VASH2 deficiency accelerated the renal tubular damage in I/R-induced AKI.

### 2.2. VASH2 Deficiency Promoted I/R-Induced Oxidative Stress Accumulation and Apoptosis

**Figure 2:**
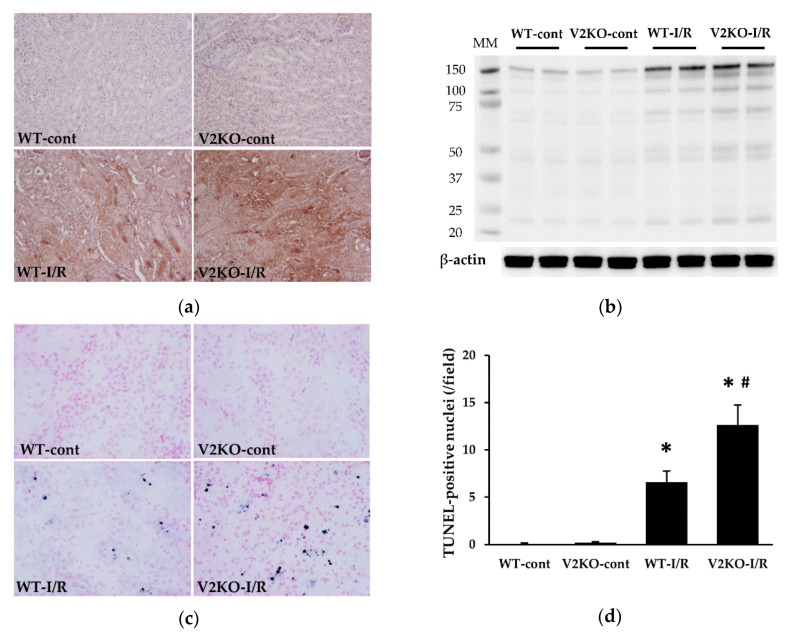
VASH2 deficiency promoted oxidative stress marker accumulation and apoptosis in I/R-induced acute kidney injury (AKI). (**a**) Immunohistochemical images of malondialdehyde (MDA) accumulation in the kidney (original magnification, ×200). (**b**) Immunoblots for 4-hydroxy-nonenal (4-HNE) and β-actin. Each lane was loaded with 40 µg of protein. MM, molecular weight. (**c**) Representative images for terminal uridine deoxynucleotidyl transferase-mediated dUTP nick-end labeling (TUNEL) staining in the kidney. The apoptotic nuclei were stained blue. (**d**) A larger number of TUNEL-positive nuclei were found in V2KO-I/R mice than in WT-I/R mice. *n* = 6 for each group. * *p* < 0.01 vs. WT-cont, ^#^ *p* < 0.05 vs. WT-I/R. Each column shows the mean ± SD.

Consistent with renal tubular injury, oxidative stress markers were accumulated in the kidney after I/R injury. Immunohistochemistry revealed the accumulation of the representative lipid peroxide, malondialdehyde (MDA), in injured renal tubules (Figure 2a), and immunoblotting showed increased renal levels of another oxidative stress marker, 4-hydroxy-nonenal (4-HNE), in I/R injury (Figure 2b). The accumulation of MDA and 4-HNE was more prominent in V2KO-I/R mice than in WT-I/R mice (Figure 2a,b). Apoptotic cells in the kidney were evaluated by terminal uridine deoxynucleotidyl transferase-mediated dUTP nick-end labeling (TUNEL) staining. A significantly larger number of apoptotic cells were detected in V2KO-I/R mice than in WT-I/R mice (Figure 2c,d).

### 2.3. VASH2 Deficiency Enhanced Neutrophil Infiltration in I/R-Induced Renal Tubular Injury

**Figure 3:**
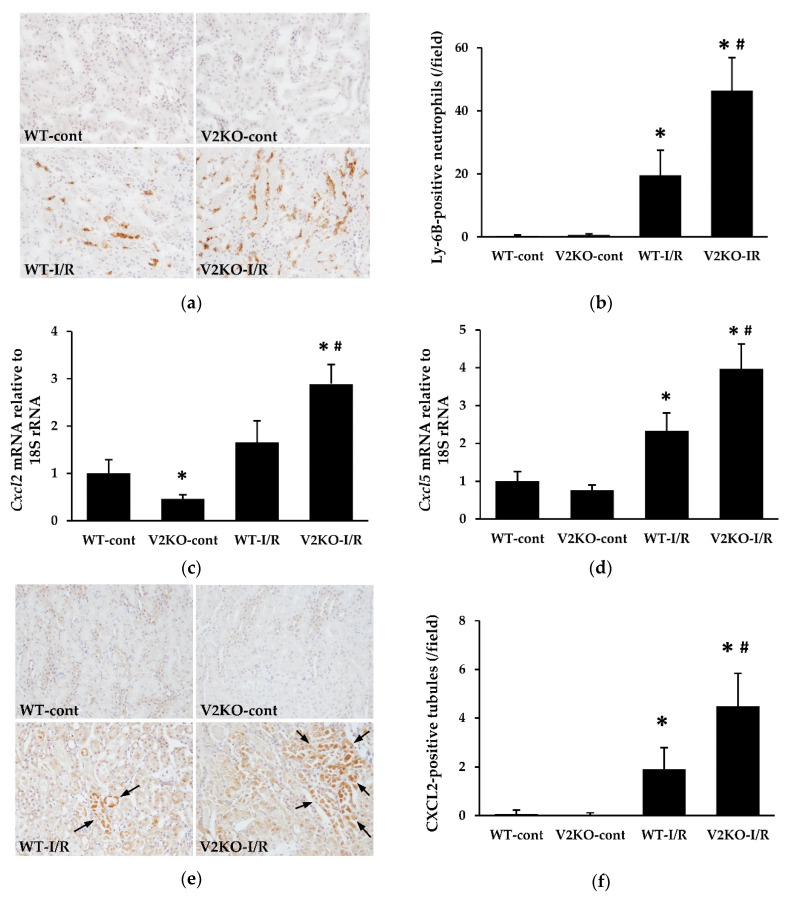
VASH2 deficiency accelerated neutrophil infiltration in I/R-induced kidney injury. (**a**) Immunohistochemical images of Ly-6B.2, a marker for neutrophils, in the kidney (original magnification, ×200). (**b**) The number of Ly-6B.2-positive cells was higher in V2KO-I/R mice than WT-I/R mice; (**c**) *Cxcl2* and (**d**) *Cxcl5* mRNA expression in the kidney. Data were normalized with the expression of 18S rRNA. (**e**) Immunohistochemical images of CXCL2 (original magnification, ×200). CXCL2-positive tubules are indicated by arrows. (**f**) The number of CXCL2-positive tubules was higher in V2KO-I/R mice than WT-I/R mice. *n* = 6 for each group. * *p* < 0.01 vs. WT-cont, ^#^ *p* < 0.05 vs. WT-I/R. Each column shows the mean ± SD.

Neutrophil infiltration is known to be an important contributor to the development of I/R-induced renal tubular injury. The number of Ly-6B.2-positive neutrophils in the kidney was examined. Neutrophil infiltration was significantly increased by I/R injury in WT mice, whereas the increase in infiltration was more prominent in V2KO-I/R mice than in WT-I/R mice (Figure 3a,b). The expression of two major neutrophil chemoattractants, CXCL2 and CXCL5, was increased by I/R injury, and the increase in expression of these chemokines was higher in V2KO-I/R mice than in WT-I/R mice (Figure 3c,d). Immunohistochemistry revealed increased CXCL2 expression in injured renal tubular epithelium (Figure 3e) with the number of CXCL2-positive tubules being significantly higher in V2KO-I/R mice than WT-I/R mice (Figure 3f).

### 2.4. VASH2 Deficiency Accelerated PTC Loss in I/R-Induced Kidney Injury

**Figure 4:**
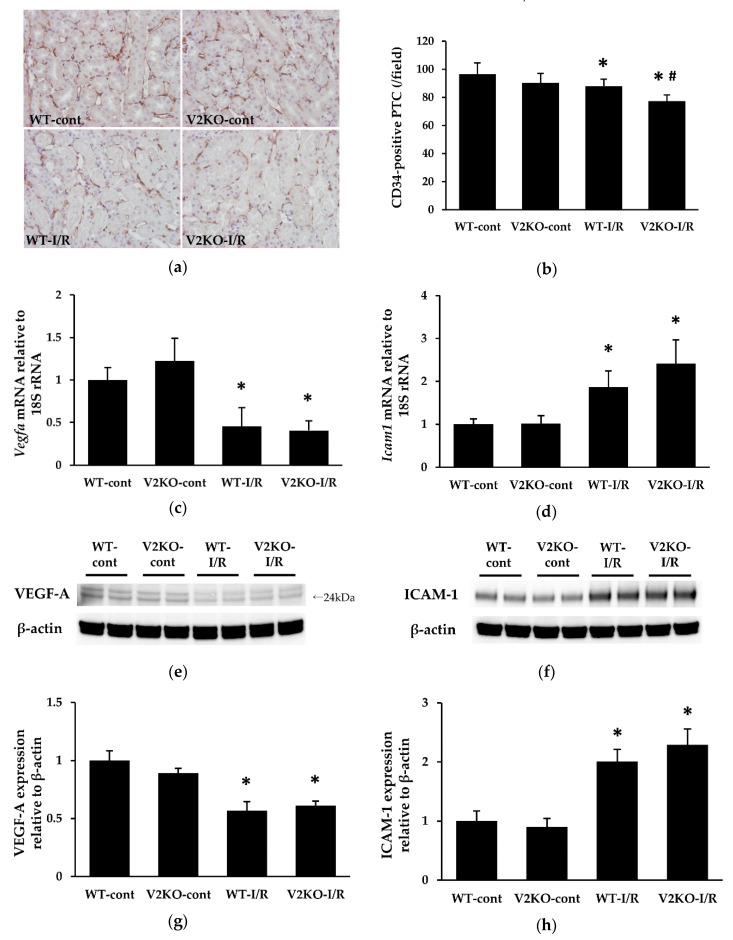
VASH2 deficiency accelerated I/R-induced peritubular capillary (PTC) loss in the kidney. (**a**) Immunohistochemical images of the endothelial marker CD34 in the kidney (original magnification, ×200). (**b**) The decrease in the number of CD34-positive PTCs was greater in V2KO-I/R mice than WT-I/R mice; (**c**) *Vegfa* and (**d**) *Icam1* mRNA expression in the kidney. Data were normalized with expression of 18S rRNA. (**e**,**f**) Immunoblots to quantify protein levels of VEGF-A and ICAM-1, respectively. VEGF-A bands were observed at 24 kDa. Each lane was loaded with 40 µg of protein. (**g**,**h**) Densitometric analysis of the immunoblots. Data were normalized to β-actin. *n* = 6 for each group. * *p* < 0.01 vs. WT-cont, ^#^ *p* < 0.05 vs. WT-I/R. Each column shows the mean ± SD.

PTCs are essential for normal tubular function as they deliver oxygen and nutrients to tubular epithelial cells. I/R-injury-induced PCT loss has been shown in previous studies [18]. In the present study, the number of CD34-positive PTCs was significantly decreased by I/R injury in WT mice. Such PTC loss was more prominent in V2KO mice than WT mice (Figure 4a,b). Renal VEGF-A expression was also significantly decreased by I/R injury. However, there were no differences in VEGF-A mRNA levels between WT-I/R and V2KO-I/R mice (Figure 4c). I/R-injury-induced PTC damage is known to be associated with increased expression of intercellular adhesion molecule-1 (ICAM-1). Here, renal ICAM-1 expression was upregulated by I/R injury. Although the increase in ICAM-1 expression tended to greater in V2KO-I/R mice than that in WT-I/R mice, the difference was not statistically significant (Figure 4d). The protein levels of VEGF-A (Figure 4e,g) and ICAM-1 (Figure 4f,h) followed the same tendency as their respective mRNA levels, and there were no significant differences in protein levels of either VEGF-A or ICAM-1 between V2KO-IR and WT-I/R mice.

### 2.5. Endogenous VASH2 Expression in I/R-Induced Kidney Injury

**Figure 5:**
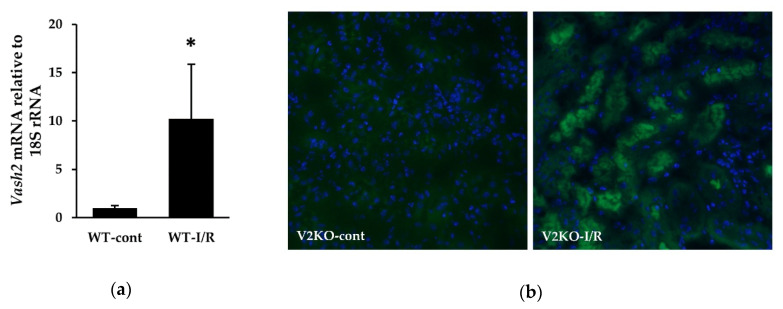
Endogenous VASH2 expression in the kidney in response to I/R injury. (**a**) *Vash2* mRNA levels determined by real-time PCR in the kidneys from WT-cont and WT-I/R mice. *n* = 6 for each group. Data were normalized with the expression of 18S rRNA. (**b**) Immunofluorescence staining for β-galactosidase (β-gal) to detect β-gal expression in the kidneys from V2KO-cont (left) and V2KO-I/R (right) mice. For nuclear staining, 4′,6-diamidino-2- phenylindole (DAPI) was used (original magnification, × 200). * *p* < 0.01 vs. WT-cont. The column shows the mean ± SD.

Renal *Vash2* mRNA expression in WT-cont and WT-I/R mice was examined by real-time PCR. I/R injury remarkably increased *Vash2* expression in the kidney (Figure 5a). As β-galactosidase (β-gal) gene was inserted into the allele of *Vash2* gene in V2KO (*Vash2*^LacZ/LacZ^) mice [19], the localization of β-gal under the control of *Vash2* promoter was identified by immunostaining of β-gal in the kidney from V2KO mice. β-gal-positive renal tubular epithelial cells were not detected in the kidney without I/R injury, whereas a large number of β-gal-positive renal tubules was observed after I/R injury (Figure 5b). These results suggested that VASH2 expression was markedly upregulated in renal tubular epithelial cells in response to I/R-induced renal injury.

## 3. Discussion

In the present study, genetic deletion of a novel proangiogenic factor VASH2 resulted in exacerbation of renal I/R-induced kidney injury, with enhanced neutrophil infiltration and PTC loss. VASH2 expression was markedly upregulated by I/R injury-induced renal tubular damage, suggesting that increased expression of VASH2 was essential for alleviating acute tubular damage in renal I/R injury.

Renal I/R injury is a common etiology in human AKI, such as in kidney transplantation. Its pathogenesis has long been investigated in experimental and clinical studies. Ischemia reduces blood flow to renal tubular epithelial cells, especially in the outer medulla, and reperfusion can increase oxidative stress, leading to renal tubular cell apoptosis, as shown in Figure 1 and Figure 2. In such processes, mitochondrial dysfunction might contribute to generate high levels of reactive oxygen species (ROS), which are necessary to initiate and maintaining renal tubular injury. Since excessive mitochondrial ROS production induces peroxidation of unsaturated fatty acids, increased accumulation of lipid peroxidation products, including 4-HNE and MDA, in the kidney might reflect the onset of both oxidative stress and mitochondrial dysfunction in AKI. Therefore, the detection of higher levels of these lipid peroxidation products in the kidney of V2KO-I/R mice suggests that VASH2 protects mitochondria from I/R injury. However, recent evidence has revealed the importance of inflammation and microvascular damage in the progression of renal I/R injury. The earliest population of the inflammatory cells that infiltrate into the renal interstitium is neutrophils [20]. Neutrophil infiltration can be induced by increased expression of neutrophil chemoattractants, such as CXCL1, CXCL2, and CXCL5. These chemokines have been demonstrated to be upregulated in renal tubular cells in response to I/R injury [21]. However, microvascular damage also contributes to the inflammatory cell infiltration. Renal I/R-injury-induced endothelial damage results in PTC rarefaction and accelerates reduced blood flow to renal tubular cells. Concurrently, I/R injury is known to upregulate ICAM-1 expression in the PTC endothelial cells, leading to the promotion of neutrophil adhesion and migration into the renal interstitium [22]. Thus, inflammation and endothelial damage are closely related, and both contribute to the progression of renal I/R injury.

VASH2 is a novel proangiogenic factor. It was demonstrated that exogenous VASH2 persistently increased vascularity, whereas VASH2 deficiency resulted in a lower vascular density in a murine subcutaneous angiogenesis model [19]. In addition, VASH2 expression was associated with accelerated angiogenesis in human ovarian cancer [14], and downregulation of VASH2 in endometrial cancer cells inhibited tumor angiogenesis [23]. VASH2 was originally identified as a homolog of VASH1 [13], an endothelium-derived antiangiogenic factor. However, contrary to VASH1, the main source of VASH2 does not seem to be endothelial cells. Notably, VASH2 has been reported to be highly expressed in embryonic stem cells and induced pluripotent stem cells [24], whereas it is scarcely expressed in various types of differentiated cells, including epithelial and endothelial cells. Therefore, the physiological roles of VASH2 remain to be elucidated. Although VASH1 and VASH2 have opposite effects on tumor angiogenesis, they may not always exert opposite functions in various pathological processes. VASH1 is constitutively expressed in endothelial cells, and it has been shown that VASH1 expression is important to enhance cellular stress tolerance [25]. We recently reported that heterozygous deficiency of VASH1 exacerbated cisplatin-induced AKI through PTC damage and inflammatory infiltration [26]. This report, along with the present study, suggests that both VASH1 and VASH2 are required to prevent the progression of AKI.

As mentioned above, VASH2 is barely expressed in differentiated cells. Therefore, almost all tissues and organs, including the kidney, display extremely low VASH2 expression. However, once normal differentiated cells are transformed into cancer cells, such cell populations acquire the ability to highly express VASH2 [14,27]. In addition, VASH2 expression has been associated with cancer progression and poor prognosis in various malignancies, such as pancreatic and esophageal cancers [28,29]. Therefore, most cells may have the potential to upregulate VASH2 expression during cellular transformation. In our recent report, we showed that renal VASH2 expression was significantly increased in a diabetic nephropathy mouse model [16]. Furthermore, in the kidney specimens from patients with CKD, immunohistochemical analysis demonstrated that renal tubular staining for VASH2 was detected in the patients but not in the control subjects [17]. Consistent with these findings, our current findings also revealed that I/R injury markedly induced VASH2 expression in the renal tubules, suggesting that the injured tubular cells might be transformed to upregulate VASH2. Unfortunately, the regulatory mechanisms of VASH2 expression are yet to be elucidated. Although VASH1 was shown to inhibit endothelial proliferation partially through the degradation of the hypoxia-inducible factor (HIF)-1α in vitro [30], an association between VASH2 and HIF-1α has not been clearly demonstrated. However, considering that hypoxia can promote cancer angiogenesis and EMT [31], enhanced VASH2 expression in cancer cells may occur in response to hypoxia. Similarly, ischemic insult in the kidney may also induce VASH2 expression in tubular cells, and renal I/R injury may specifically upregulate VASH2 compared with other etiologies of AKI. Such hypotheses should be explored in future research.

Recently, a study demonstrated that renal tubules-specific deletion of VEGF-A led to a markedly reduced PTC area, suggesting that VEGF-A expressed in renal tubular cells is important for maintaining normal PTC structure and function [10,32]. Since VASH2 is also a proangiogenic factor, renal tubule-derived VASH2 may protect PTCs from various insults, similar to VEGF-A. Here, VASH2 deficiency resulted in prominent I/R-injury-induced PTC loss, although there were no differences in renal VEGF-A levels between WT-I/R and VASH2-I/R mice. In various gastric cancer cell lines, the mRNA expressions of VASH2 has been reported to be inconsistent with that of VEGF [33]. Thus, VASH2 may promote angiogenesis independently of VEGF. As mentioned above, renal ischemic insults upregulate ICAM-1 expression in PTC endothelial cells, leading to increased neutrophil infiltration into the renal interstitium. In this study, VASH2 deficiency mice displayed a higher number of infiltrated neutrophils in the kidney than in WT mice, and although renal ICAM-1 expression tended to be higher in V2KO-I/R mice than WT-I/R mice, the difference was not statistically significant. These results suggested that the increased expression of chemoattractants in the renal tubular cells, rather than higher ICAM-1 expression triggered by endothelial damage, was responsible for a more prominent neutrophil infiltration in V2KO-I/R mice.

Taken together, renal VASH2 expression has some protective effects in I/R injury-related AKI. However, VASH2 is not always good for the kidney. In a diabetic nephropathy mouse model, VASH2 deficiency ameliorated albuminuria and glomerular injury [16]. In addition, recent evidence suggested that VASH2 not only promotes tumor angiogenesis but also induces EMT in cancer cells, thus enhancing the malignant behavior. EMT is a process that is involved in the dedifferentiation of mature epithelial cells. Overexpression of VASH2 in human hepatocellular carcinoma cells induced the expression of the EMT-related nuclear factor, zinc finger E-box-binding homeobox 2 (ZEB2), and the mesenchymal marker vimentin [15], whereas knockdown of VASH2 in ovarian cancer cells inhibited the expression of ZEB2 and mesenchymal markers [34]. Considering the pathological roles of EMT in renal fibrosis, sustained expression of VASH2 in renal tubular epithelial cells after I/R injury may accelerate post-AKI interstitial fibrosis. Although VASH2 exerts protective effects on I/R injury in the acute phase, it may paradoxically exert adverse effects during the recovery phase. Thus, based on the results of this study, whether a therapeutic strategy that enhances VASH2 expression in tubular epithelial cells might be effective for I/R injury-induced AKI remains undetermined.

This study has a limitation that whole body, but not conditional, V2KO mice were used in the animal experiments. Since increased VASH2 expression was observed in renal tubular cells in WT-I/R mice (Figure 5), we believed that VASH2 deficiency in renal tubular epithelial cells was closely associated with the exacerbation of renal I/R injury. However, other minor source-derived VASH2 might affect the results in this study. Considering that VASH2 was found be localized in bone-marrow-derived mononuclear cells in a skin-flap angiogenesis model [19,35], inflammatory cells may be a minor source of VASH2 in an I/R model, although mononuclear cells are hardly detected in the acute phase of renal I/R injury. Since it is currently impossible for us to use conditional V2KO mice, whether such minor-source-derived VASH2 affected the results could not be determined in this study. Another potential limitation of this study is the small number of mice used for each group. Although WT-I/R and V2KO-I/R mice displayed significant differences in renal injury, more replicates might be needed to clarify the underlying mechanisms by which VASH2 exerted protective effects in AKI, including ICAM-1 expression.

In conclusion, increased expression of proangiogenic factor VASH2 may be essential to prevent the progression of renal tubular injury in I/R-induced AKI, possibly through protecting PTCs and preventing inflammatory infiltration. Although further studies are needed to clarify the physiological and pathological roles of VASH2 in the kidney, VASH2 expression may be a novel target for the acute phase of AKI.

## 4. Materials and Methods

### 4.1. Animals and Experimental Protocols

C57BL/6J *Vash2* homozygous knockout (*Vash2*^LacZ^/^LacZ^; V2KO) mice were obtained from the Institute of Development, Aging, and Cancer, Tohoku University (Sendai, Japan). The experimental protocol was approved by the Animal Care and Use Committee in Okayama University (Okayama, Japan; approval number OKU-2014509 [17/10/2014] and OKU-2017345 [24/08/2017]). Eight-week-old male C57BL/6J WT and V2KO mice were fed a standard pellet laboratory chow and provided with water ad libitum. Renal I/R injury in WT and V2KO mice was induced by clipping bilateral renal pedicles with plastic clips for 25 min. Control WT and V2KO mice received sham operation. Twenty-four hours later, blood samples were collected from the inferior vena cava of mice under anesthesia, and then the kidneys were harvested. The experimental subgroups included (1) control WT (WT-cont), (2) control V2KO (V2KO-cont), (3) WT with I/R injury (WT-I/R), and (4) V2KO with I/R injury (V2KO I/R) mice (*n* = 6 in each group). Serum creatinine and BUN concentrations were measured by FUJIFILM Monolith Co., Ltd. (Tokyo, Japan).

### 4.2. Histological Analysis

For histological analysis, 10% buffered formalin-fixed and paraffin-embedded 4 µm sections were stained with PAS for observation by light microscopy. Renal tubular injury was quantified as ATN score, defined as the percentage of renal tubules with detachment of epithelium, loss of brush border, or cast formation at 200× magnification, as follows: 0 = none, 1 = <10%, 2 = 10–25%, 3 = 26–50%, 4 = 51–75%, and 5 = >75% [36]. The kidney sections were evaluated by two investigators (H.M. and K.T.) in a blinded manner.

### 4.3. Immunohistochemistry

Kidney sections (4 µm) fixed with 10% buffered formalin and embedded in paraffin were used for MDA, CXCL2, and CD34 immunohistochemistry, as previously described [37,38]. After deparaffinization with xylene and rehydration with ethanol, the sections were treated with 3% H_2_O_2_ for 10 min to inactivate endogenous peroxidase activity. For CD34 staining, the sections were then treated with 10 mM citrate buffer (pH 6.0) for 10 min in a microwave for antigen retrieval. After incubation with serum-free Protein Block (Dako, Glostrup, Denmark) for 30 min, the sections were incubated with mouse monoclonal anti-MDA (JaICA, Fukuroi, Shizuoka, Japan), rabbit anti-CXCL2 (Abcam, Cambridge, UK), and rat anti-CD34 (Abcam, Cambridge, UK) antibodies overnight at 4 °C. Sections were then incubated with MACH2 Mouse or Rabbit HRP-Polymer (Biocare Medical, Pacheco, CA, USA) for MDA and CXCL2, respectively, and biotinylated anti-rat IgG (Vector Laboratories, Burlingame, CA, USA) followed by VECTASTAIN Elite ABC Kit (Vector laboratories, Burlingame, CA, USA) for CD34. ImmPACT DAB (Vector Laboratories, Burlingame, CA, USA) was used as a chromogen. The nuclei were counterstained with hematoxylin. The number of CXCL2-positive tubules was counted at 200 × magnification in ten fields of view per section. The number of CD34-positive PTCs was counted at 200 × magnification in ten fields of view per section.

For immunohistochemical detection of neutrophils, 4 µm frozen kidney sections fixed with methanol were used. After endogenous peroxidase inactivation, the sections were incubated with anti-Ly-6B antibody (Bio-Rad, Hercules, CA, USA) overnight at 4 °C, followed by incubation with HRP-conjugated anti-rat IgG (Vector laboratories, Burlingame, CA, USA) for 60 min at room temperature. ImmPACT DAB was then applied, followed by counterstaining with hematoxylin. The number of Ly-6B-positive neutrophils was counted at 200 × magnification in ten fields of view per section.

### 4.4. Apoptosis Detection

TACS 2 TdT-Blue Label In Situ Apoptosis Detection Kit (Trevigen, Gaithersburg, MD, USA) was used to detect TUNEL-positive apoptotic cells in 8 µm frozen sections of the kidneys, according to the manufacturer’s protocol [39]. The nuclei were counterstained with Nuclear Fast Red. Ten fields of view per section at 200 × magnification were evaluated to determine the average number of apoptotic cells.

### 4.5. Immunofluorescence

Immunofluorescence was performed on 4 µm frozen kidney sections fixed with methanol, as previously described [16,40]. After treatment with phosphate-buffered saline containing 0.1% Tween 20 for penetration, the sections were incubated with anti-β-gal antibody (Promega, Madison, WI, USA) overnight at 4 °C, followed by incubation with Alexa Fluor 488-labeled secondary antibody (Thermo Fischer Scientific, Waltham, MA, USA). DAPI (Millipore, Burlington, MA, USA) was used for nuclear staining. The images were obtained by fluorescence microscopy (BZ-8000; Keyence, Osaka, Japan).

### 4.6. Immunoblotting

Kidney tissues containing cortex and medulla were homogenized in RIPA lysis buffer (Santa Cruz Biotechnology, Santa Cruz, CA, USA) at 4 °C. Cultured cell lysates were prepared as described below. Extracted protein samples were separated by sodium dodecyl sulfate-polyacrylamide gel electrophoresis and transferred onto nitrocellulose membranes [16]. The membranes were incubated overnight at 4 °C with the following primary antibodies: (1) anti-4-HNE antibody (JaICA, Fukuroi, Shizuoka, Japan), (2) rabbit anti-VEGF-A antibody (Proteintech, Rosemont, IL, USA), (3) rabbit anti-ICAM-1 antibody (Proteintech, Rosemont, IL, USA), and (4) anti-β-actin antibody (Sigma-Aldrich, St Louis, MO, USA). Then, they were incubated with HRP-labeled anti-mouse or anti-rabbit IgG antibody (Cell Signaling Technology, Danvers, MA, USA) for 60 min, and signals were detected using ECL Western Blotting Detection Reagents (GE Healthcare, Buckinghamshire, UK). Images were obtained with ImageQuant LAS 4000 (GE Healthcare, Buckinghamshire, UK). Band density was determined by densitometry using the ImageJ software (version 1.51) and expressed relative to the density of β-actin.

### 4.7. Real-Time PCR

RNA was extracted from the homogenized kidney tissues using RNeasy Mini Kit (Qiagen, Chatsworth, CA, USA) according to the manufacturer’s protocol and reverse transcribed into cDNA with random primers and SuperScript II Reverse Transcriptase (Invitrogen, Carlsbad, CA, USA). cDNA was added to Fast SYBR Green Master Mix (Applied Biosystems, Foster City, CA, USA) with specific oligonucleotide primers for the detection of mRNA levels, as previously described [26,39]. Quantitative real-time PCR was performed on the StepOnePlus Real-Time PCR System (Applied Biosystems, Foster City, CA, USA). The amount of PCR products was normalized with 18S rRNA. The following oligonucleotide primers were used: mouse *Cxcl2*, 5′-GCC AAG GGT TGA CTT CAA GAA CA-3′ (forward) and 5′-AGG CTC CTC CTT TCC AGG TCA-3′ (reverse); mouse *Cxcl5*, 5′-TGA TCC CTG CAG GTC CAC A-3′ (forward) and 5′-CTG CGA GTG CAT TCC GCT TA-3′ (reverse); mouse *Vegfa*, 5′-ACA TTG GCT CAC TTC CAG AAA CAC-3′ (forward) and 5′-TGG TTG GAA CCG GCA TCT TTA-3′ (reverse); mouse *Icam1*, 5′-AAC TGT GGC ACC GTG CAG TC-3′ (forward) and 5′-AGG GTG AGG TCC TTG CCT ACT TG-3′ (reverse); mouse *Vash2*, 5′-GGC TAA GCC TTC AAT TCC CC-3′ (forward) and 5′-CCC ATT GGT GAG ATA GAT GCC-3′ (reverse); mouse 18S rRNA, 5′-ACT CAA CAC GGG AAA CCT CA-3′ (forward) and 5′-AAC CAG ACA AAT CGC TCC AC-3′ (reverse).

### 4.8. Statistical Analysis

All values were expressed as means ± SD. Kruskal–Wallis test followed by post-hoc Dunn’s multiple comparison test was used for inter-group comparisons of multiple variables. The JMP 10 software (SAS Institute Inc, Cary, NC, USA) was used for statistical analyses. *p* < 0.05 was regarded as statistically significant.

## Abbreviations

AKI	Acute kidney injury
VASH2	Vasohibin-2
CKD	Chronic kidney disease
I/R	Ischemic reperfusion
NOS	Nitric oxide synthase
PTC	Peritubular capillary
VEGF	Vascular endothelial growth factor
WT	Wild-type
V2KO	VASH2 knockout
ATN	Acute tubular necrosis
MDA	Malondialdehyde
4-HNE	4-hydroxy-nonenal
TUNEL	Terminal uridine deoxynucleotidyl transferase-mediated dUTP nick-end labeling
CXCL	CXC chemokine ligand
ICAM-1	Intercellular adhesion molecule-1
β-gal	β-galactosidase
ROS	Reactive oxygen species
EMT	Epithelial-to-mesenchymal transition
ZEB2	zinc finger E-box-binding homeobox 2
